# Patient Preference for Antiepileptic Drugs Treatment in China: Evidence From the Discrete Choice Experiment

**DOI:** 10.3389/fneur.2020.602481

**Published:** 2020-12-03

**Authors:** Yingjie Hua, Zhenguo Zhu, Xueying Li, Jiaoni Gong, Siqi Ding, Jiahe Lin, Xinshi Wang, Yanru Du, Niange Xia, Rongyuan Zheng, Huiqin Xu

**Affiliations:** Department of Neurology, The First Affiliated Hospital of Wenzhou Medical University, Wenzhou, China

**Keywords:** antiepileptic drugs, discrete choice experiment, preference, epilepsy, willingness to pay (WTP)

## Abstract

**Objective:** Explore Chinese patients' risk-benefit preferences and willingness-to-pay (WTP) for antiepileptic drugs (AEDs) treatment through the discrete choice experiment (DCE).

**Method:** Six attributes including the efficacy of AEDs, adverse reactions (digestive system, neuropsychic systems, and the effects on the fetus), dosing frequency and drug costs (to estimate patient WTP) were included in the DCE questionnaire based on results collected from literature reviews, expert consultation, and patient survey. The alternative-specific conditional logit model was used to analyze patient preference and WTP for each attribute and its level and to assess the sociodemographic impact and clinical characteristics.

**Results:** A total of 151 valid questionnaires were collected. The result shows that five out of the six attributes are significant, except the dosing frequency. Among the six attributes, the efficacy of AEDs (10.0; 95% CI 8.9–11.1) is mostly concerned by patients, followed by the effects of AEDs on the fetus (8.9; 95% CI 7.7–10.1), duration of side effects in the neuropsychic system (4.9; 95% CI 3.7–6.0) and adverse reactions of the digestive system (3.2; 95% CI 1.5–4.2). The patients surveyed are willing to spend ¥ 1,246 (95% CI, ¥ 632- ¥ 1,861) per month to ensure 100% seizure control, and ¥ 1,112 (95% CI, ¥ 586–¥ 1,658) to reduce the risk of the drug affecting the fetus to 3%. Besides, it was found that personal characteristics including the intention for conception and AEDs treatment regimens have statistical significance.

**Conclusion:** Improving the drug's efficacy and reducing its side effects are predominant considerations for patients with epilepsy in China, especially for those who are concerned about the seizure control and the drug effect on the fetus. This finding is useful to physicians and can encourage shared decision-making between the patients and their doctors in the clinic.

## Introduction

Epilepsy is a chronic brain disorder with multiple etiologies. Its primary pathology is a recurrent, paroxysmal, and transient brain dysfunction caused by excessive discharge of cerebral neurons. The incidence rate of epilepsy was 61.44 per 100,000 person-years, and about 70 million people worldwide suffer from this disease (1–3). More than nine million people have epilepsy in China, and about two-thirds of them have active one. What's more, 400,000 new cases of epilepsy are diagnosed in China annually (4). AEDs is the cornerstone of epilepsy management and it helps to reduce the severity and seizures frequency.

Additionally, patients' treatment adherence depends on many factors, including efficacy, adverse effects, safety, ease of use, and costs, especially the adverse effects like nausea, vomiting, dizziness, tremor, skin rash, memory problems, and cognitive impairment (5–7). Furthermore, drugs' seizures and adverse effects can affect health-related quality of life (HRQOL) and patients' satisfaction (8). In practice, drug selection is mainly determined by the doctor without considering patients' opinions that mattered. Hence, doctors need to understand patients' preferences for AEDs and consider patients' opinions when making clinical practice decisions so as to improve treatment adherence, patient's life quality, and epilepsy control.

Discrete choice experiments (DCEs) are effective techniques for eliciting preferences. Hypothesis scenarios (such as scheme A and scheme B) are presented through different attributes and attribute levels, requiring participants to choose their favorite opinions and quantify each different attribute's individual impact on decision-making (9–17). DCEs have been widely used in many fields, such as marketing, transport economics, and environmental economics (18, 19). Besides, DCEs are increasingly important in health economics (20). They have been used to quantify the preference of patients or doctors for treatments on diseases like tumors (9–11), kidney disease (12), anxiety (13), and depression (13). In the field of epilepsy, DCEs are mainly used to study the choice of AEDs. For example, Lloyd et al. (14) and Manjunath et al. (15) used DCEs or conjoint analysis to discover patients' preference and willingness to pay (WTP) of AEDs or add on AEDs. Ettinger et al. (16) conducted a DCE to find the difference between neurologists and patients in choosing AED. Holmes et al. (17) combined the SANAD clinical trial data to estimate the utility of hypothetical AEDs.

However, studies assessing the patient's preference for AEDs were mainly conducted in developed countries such as the U.S. and the U.K., while few have been done in developing countries. China is a developing country with about 6 million people having active epilepsy, and most of them need long-term AEDs treatment. Therefore, revealing patients' preference for AEDs through DCEs may help improve patients' treatment adherence in developing countries so as to reduce the incidence of drug-resistant epilepsy (21–24).

## Methods

### Identify Attributes and Levels

The primary step in DCEs is to determine appropriate attributes and levels (25). As many factors affect the choice of AEDs, the attributes included must represent the compelling elements of the clinical scenario and facial validity. Methods for selecting attributes include literature reviews, professional recommendations, focus groups, interviews or consultation with physicians or patients, patient surveys, expert reviews, and so on (26–29). Excessive attributes may increase the cognitive burden of responders, while insufficient attributes will make it difficult to obtain convincing results. Therefore, the number of attributes should be appropriate, usually 4–9 in the field of Medicine and Health (30).

Through literature reviews (5, 14–17, 21–23, 31–34), patient survey, and expert consultation in our center, we identified six attributes: the efficacy of AEDs, adverse reactions of the digestive system, the duration of side effects in neuropsychic systems, dosing frequency, effects of AEDs on the fetus and drug cost. This paper's drug costs were used as a value attribute to explore patient trade-off in AEDs costs. The preference of males with epilepsy was also investigated for the attribute relating to AEDs' effects on the fetus. The reason is that males with epilepsy worry that AEDs may be detrimental to the fetus. As reported in the literature, the baby's risk of congenital anomalies will increase slightly if the father uses AEDs 3 months before the mother's pregnancy (35). There are six attributes, with three levels per attribute (Table 1). For this study, all attributes and levels were identified by published literature and calibrated by neurologists.

**Table 1:** Treatment attributes and levels were chosen for the DCE.

**Attributes**	**Levels**
**Efficacy of AEDs** When you take the AED, seizure frequency can be reduced	Seizure-free (100% controlled)
	Seizure frequency is reduced by more than half (75% less)
	Seizure frequency is reduced by half (50% less)
**Adverse reactions of the digestive system** When you take the AED, you may have nausea or/and vomiting	0 in 10 people (0%) 1 in 10 people (10%) 3 in 10 people (30%)
**Duration of side effects in neuropsychic systems** Some adverse reactions of the AED will last for a few days or weeks. These side effects include drowsiness, dizziness, headache, tremor, double vision, blurred vision or irritable	None Last for 1 week Last for 4 weeks
**Dosing frequency** How often do you take the AED	Once a day Twice a day Thrice a day
**Effects of AEDs on the fetus** Taking the AED may harm your fetal, causing problems such as neural tube defect, attention-deficit/hyperactivity disorder (ADHD), and cognitive impairment	3 in 100 people (3%) 6 in 100 people (6%) 10 in 100 people (10%)
**Drug costs** The cost of this AED per month	¥ 200 per month ¥ 400 per month ¥ 600 per month

### Construction of the DCE Questionnaire

**Figure 1:**
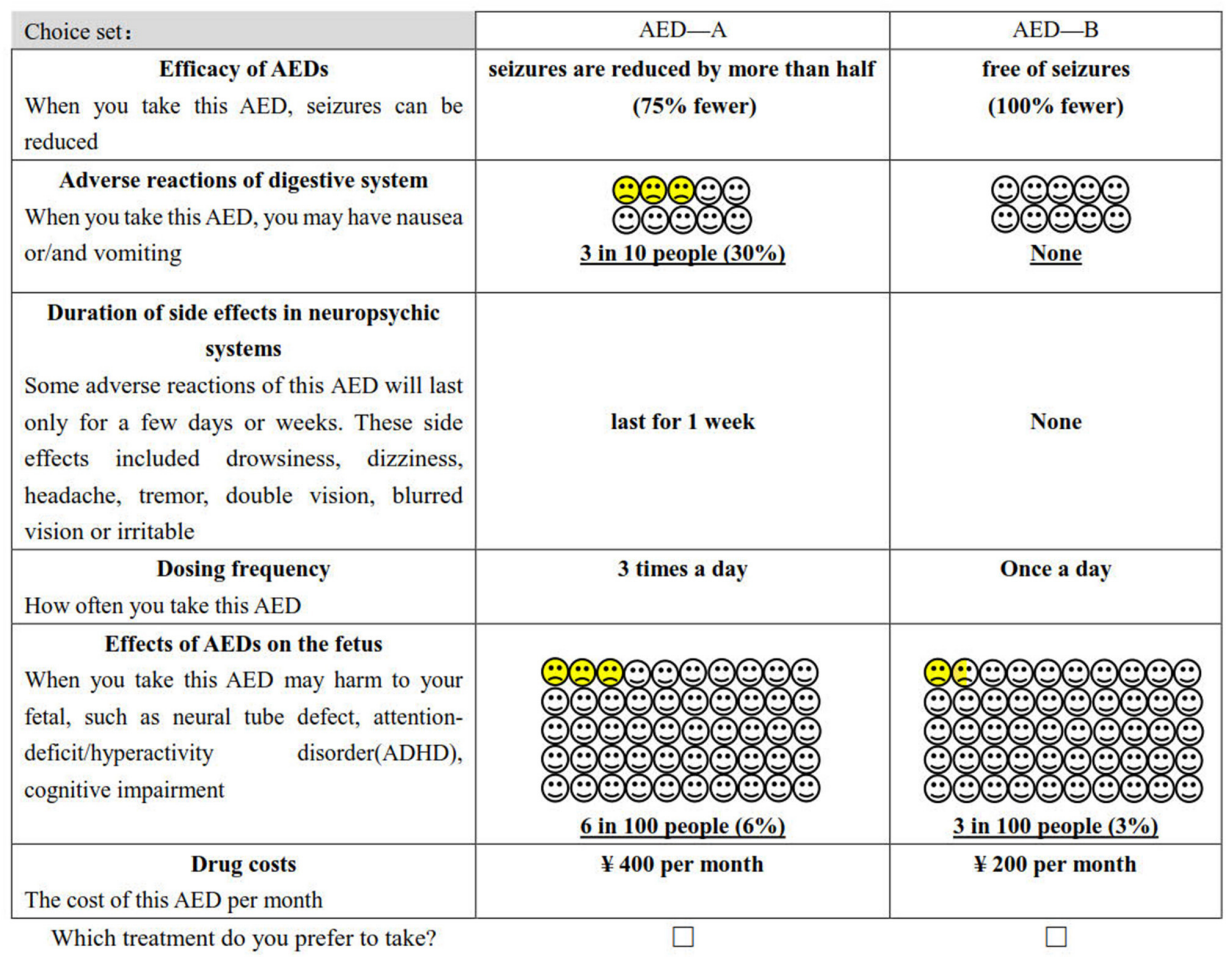
Example of the choice set in the questionnaire.

The study obtained 729 (3^6^) hypothetical scenarios by combining attributes and levels (six attributes with three levels per attribute). It is not realistic for the patient to complete all the choice sets. Therefore, we used D-optimal design (SAS 9.4, the procedure by %ChoicEff Macro) to generate the most optimal scenarios (36). The final experimental design consists of 18 choice sets (An example of a choice set is shown in Figure 1), and each respondent was required to finish 18 trade-off questions. All of them are binary choices, which means that patients must choose between drug A or drug B. In this questionnaire, the choice set that “*hypothetical” AED A was superior to B*′ was used to explore preferences consistency.

In addition to DCE, the paper also investigated the sociodemographic and patients' clinical characteristics. See Table 2 for details.

**Table 2:** Sociodemographic and clinical characteristics of the patients with epilepsy.

**Characteristics**	**Subjects (*n* = 151)**
Male/female	72/79
Age (years), mean, range	32, 19–68
Marital status, *n* (%)	
Unmarried	61 (40.4)
Married	88 (58.3)
Divorced	2 (1.3)
The length of education (years), mean, range	12, 4–23
Education status, *n* (%)	
Primary	6 (4.0)
Secondary	78 (51.7)
Tertiary	67 (44.3)
Annual household income (ten thousand yuan), *n* (%)	
<5	32 (21.2)
5–15	77 (51.0)
>15	42 (27.8)
Ready for conception	
Yes	31 (20.5)
No	120 (79.5)
Onset age (years), mean, range	22, 2–63
Duration of epilepsy (years), mean, range	10, 0–39
Epilepsy tpyes, *n* (%)	
Focal	85 (56.3)
Generalized	12 (7.9)
Combined generalized and focal	0 (0)
Unknown	54 (35.8)
Seizure frequency in recent 1 year, *n* (%)	
≥Once a week	7 (4.6)
≥Once a month and < Once a week	29 (19.2)
≥Once a year and < Once a month	51 (33.8)
No seizures	64 (42.4)
AEDs treatment regimens, *n* (%)	
Monotherapy	77 (51.0)
Combinations of two AEDs	58 (38.4)
Combinations of three or more AEDs	16 (10.6)

### Patient Recruitment

Patients were consecutively recruited from the epilepsy center of the First Affiliated Hospital of Wenzhou Medical University, China. All patients were interviewed face to face with neurologists, which may help patients to understand the questionnaire.

The inclusion criteria are as follows: (1) Patients aged 18 years or older; (2) a definite diagnosis of epilepsy according to the 2017 International League Against Epilepsy (ILAE) criteria; (3) no cognitive impairment and able to understand the questionnaire; (4) willing to participate and can provide informed consent. Patients will be excluded if they (1) have incomplete questionnaire information; (2) answer the consistency check incorrectly in the questionnaire.

From 31 May 2020 to 1 August 2020, 176 patients have consented to complete the survey. Twenty-five patients were excluded from the study as they failed to answer the consistency check correctly, meaning that they did not understand the task adequately. The final sample in our study included 151 patients. The number is large enough for reliable statistical analyses according to related studies (37).

### Statistical Analysis

Sociodemographic characteristics and clinical variables were evaluated by descriptive analysis.

The alternative-specific conditional logit model (also called McFadden's choice model) was used to analyze the data. Coefficients of the model indicate the importance of each attribute and the direction of choosing AEDs treatment (38–40). In the model, effect coding was adopted to represent a dummy variable to ensure that all attribute levels can be estimated (41). Then, segmented analyses were conducted to assess how preferences varied based on sociodemographic and clinical characteristics and calculated WTP for sub-groups. STATA statistical software was used to analyze the data (version 16.0).

## Results

### Patient Demographics and Clinical Characteristics

The demographics and clinical characteristics of the respondents were presented in Table 2. This sample consists of 151 patients: the number of female patients is 79 (52.3%). The age of the patients ranges from 19 to 68, and the average age is 32. Their average length of education time is 12 years, and most of them (96.0%) have a secondary education degree or above. 20.5% of them have conception intention. A relatively large portion of patients have focal epilepsy (56.3%), but most of them have no recent seizures (42.4%). The percentage of the patients who receive monotherapy, two AEDs, and more than two AEDs are 51.0, 38.4, and 10.6%, respectively.

### Patients Preference for the Treatment of AEDs

**Figure 2:**
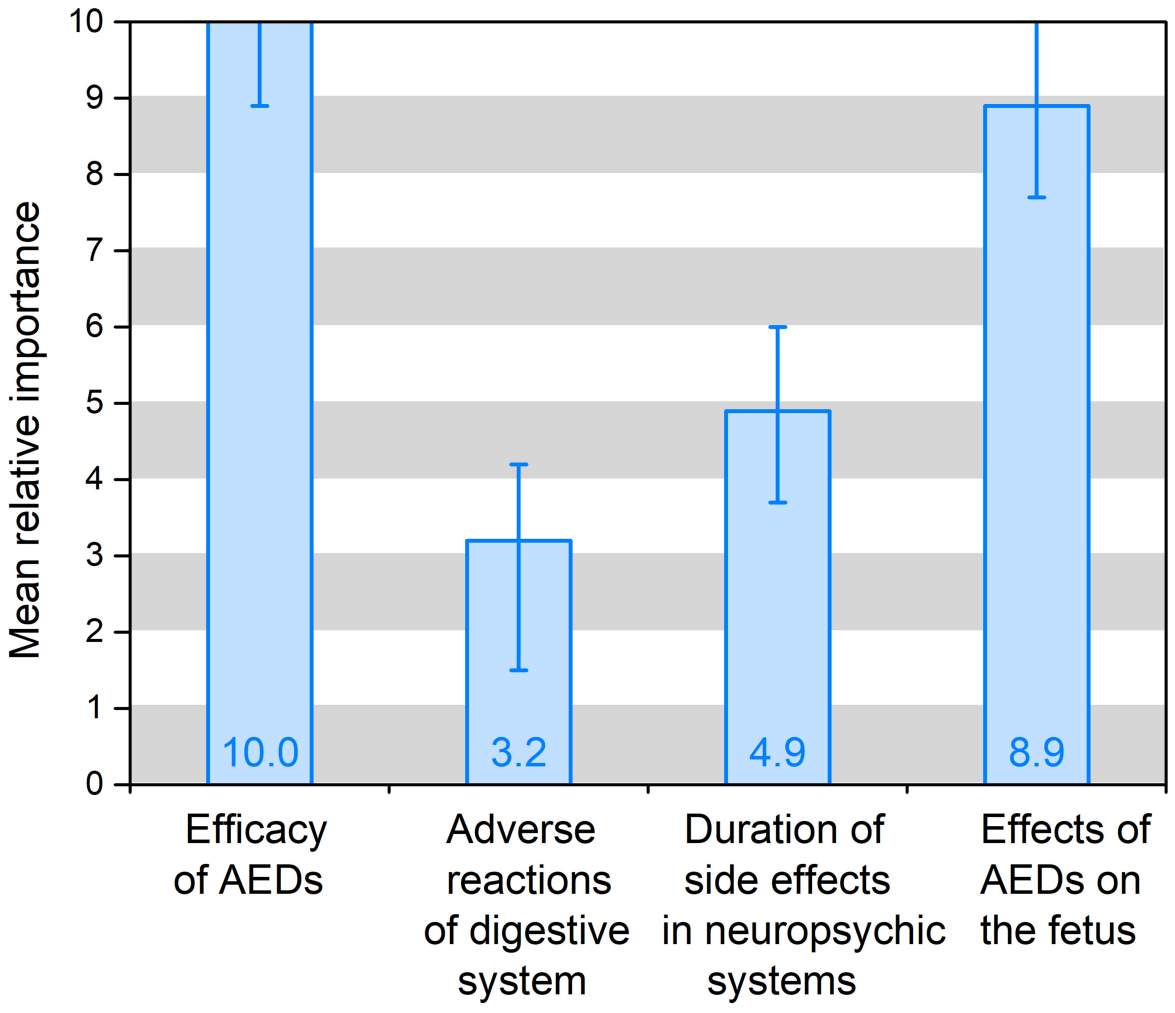
Mean relative importance for treatment of AEDs (*N* = 151).

The preference weights of the alternative-specific conditional logit model results are shown in Table 3 and Figure 2. In the model, the attribute of drug costs was used as continuous variables, and the other five as categorical variables. The results show that the coefficients are significant (*p* < 0.05) for five out of the six attributes, except the dosing frequency. That means patients' treatment preferences are not influenced by dosing frequency. Table 3 illustrates that patients prefer better seizure control and fewer side effects of drugs. What's more, they have the strongest desire for achieving 100% seizure control (coefficient = 0.778, *p* = 0.000) and lower effects (3%) on the fetus (coefficient = 0.700, *p* = 0.000). In addition, they prefer to having no adverse reactions (0%) in neuropsychic systems (coefficient = 0.378, *p* = 0.000) and in digestive system (coefficient = 0.247, *p* = 0.000). The coefficient that is <0 means that respondents have little preference for this attribute. Figure 2 shows that the mean relative importance is 95% CI, and the efficacy of AEDs is the most preferred treatment attribute (10.0; 95% CI 8.9–11.1). Apart from the efficacy of AEDs, patients rank the remaining treatment attributes as follows: effects of AEDs on the fetus (8.9; 95% CI 7.7–10.1), duration of side effects in the neuropsychic system (4.9; 95% CI 3.7–6.0), and adverse reactions of the digestive system (3.2; 95% CI 1.5–4.2).

**Table 3:** The patients' preference for treatment of AEDs: the main effects of alternative-specific conditional logit model results (*N* = 151).

		**95% CI**				
**Attributes**	Coefficient^*^	**LB**	**UB**	**SE**	***P*-value**	***Z***
**The efficacy of AEDs**						
100% controlled	0.778	0.695	0.861	0.042	0.000	18.38
75% less	−0.119	−0.197	−0.040	0.040	0.003	−2.97
50% less	−0.659	−0.745	−0.573	0.044	0.000	−15.01
**Adverse reactions of digestive system**						
0 in 10 people (0%)	0.247	0.166	0.328	0.041	0.000	5.96
1 in 10 people (10%)	0.131	0.051	0.212	0.041	0.001	3.2
3 in 10 people (30%)	−0.378	−0.454	−0.303	0.039	0.000	−9.84
**Duration of side effects in neuropsychic systems**						
None	0.378	0.287	0.470	0.047	0.000	8.1
Last for 1 week	−0.209	−0.293	−0.124	0.043	0.000	−4.82
Last for 4 weeks	−0.170	−0.243	−0.097	0.037	0.000	−4.56
**Dosing frequency**						
Once a day	−0.087	−0.175	0.001	0.045	0.052	−1.95
Twice a day	0.066	−0.009	0.140	0.038	0.085	1.72
3 times a day	0.022	−0.056	0.100	0.040	0.581	0.55
**Effects of AEDs on the fetus**						
3 in 100 people (3%)	0.700	0.602	0.798	0.050	0.000	13.97
6 in 100 people (6%)	−0.078	−0.153	−0.002	0.039	0.004	−2.02
10 in 100 people (10%)	−0.622	−0.705	−0.540	0.042	0.000	−14.84
**Drug costs**	−0.001	−0.001	−0.000	0.000	0.000	−3.94

* *The coefficient refers to the change in the respondent's utility within a given level of a attribute*.

*LB, low bound; UB, upper bound*.

### The Impact of Fertility Preparation on Treatment Preferences

**Figure 3:**
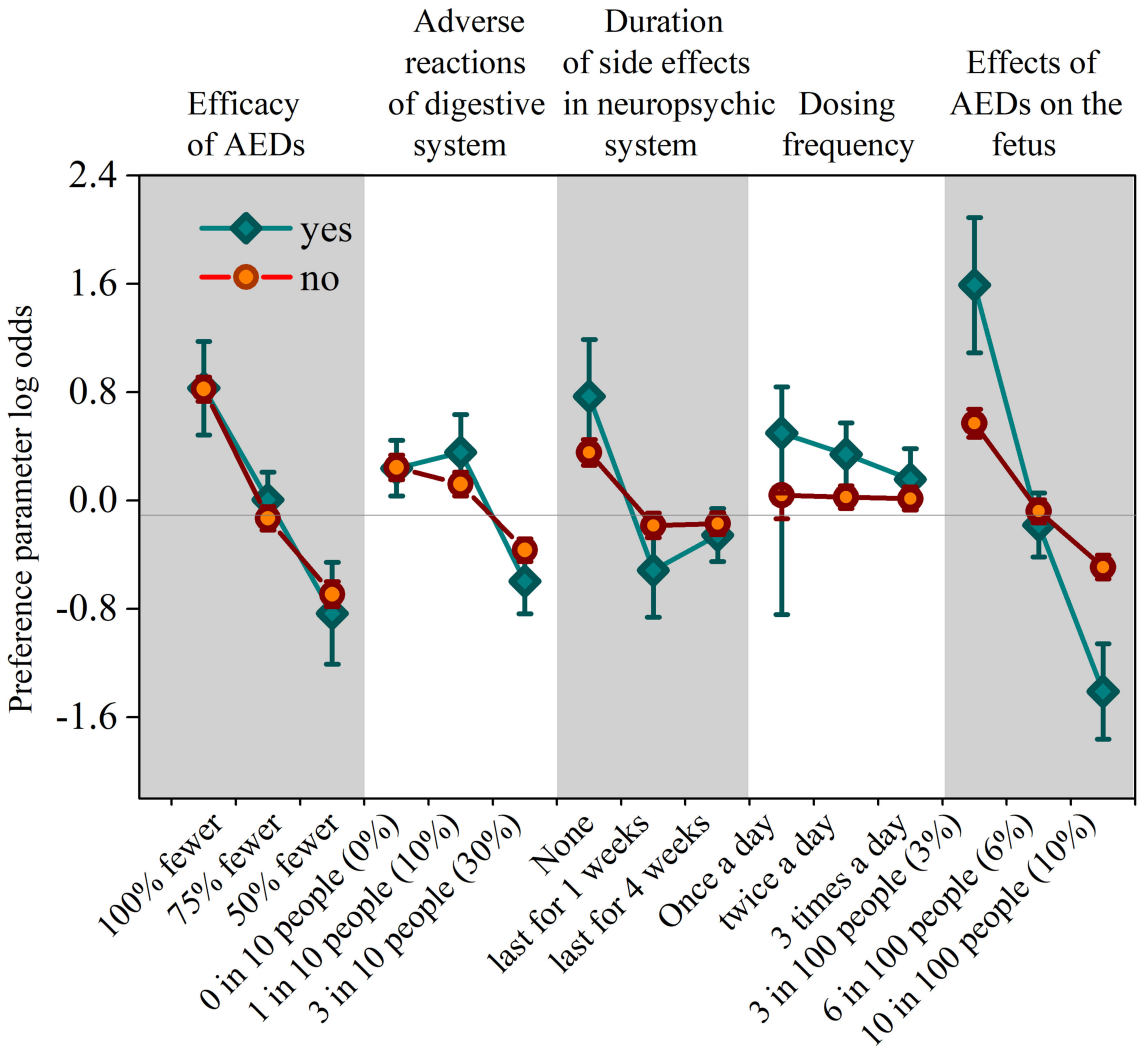
The coefficient of personal attribute in the intention for having babies. Vertical bars denote 95% confidence intervals.

After analyzing the interaction terms between patients' sociodemographic or clinical characteristics and their preferences for different attributes, the authors found that the personal attribute of pregnancy intentions is significant (*P* < 0.05). As the preference-weight shown in Figure 3, patients who have pregnancy intention have a significantly high coefficient (coefficient = 1.591, 95% CI 1.092–2.090, *p* = 0.000) in lower effects (3%) on the fetus, which is more than the double of that of the patients who are not (coefficient = 0.571, 95% CI 0.467–0.675, *p* = 0.000). This result indicates that patients with pregnancy intentions worry more about the effect of AEDs on the fetus than the efficacy of AEDs. Besides, there is little difference between patients who are trying for babies and patients who are not in the coefficients of the other four attributes.

### To Estimate the Willingness-to-Pay (WTP)

Physicians can evaluate the economic value of the treatment attributes by analyzing the attribute of drug costs. The comparison of each attribute is demonstrated in the economic value-form, which is easier for doctors to understand.

Table 4 presents the patients' trade-off of drug costs for the treatment of AEDs in each attribute. Among all respondents' WTP, patients are willing to spend ¥ 1,246 (95% CI, ¥ 632–¥ 1,861) per month to ensure 100% seizure control and ¥ 1,112 (95% CI, ¥ 586–¥ 1,658) to reduce the risk of drug effects on the fetus to 3%. The value for no side effects in neuropsychic systems is ¥ 606 (95% CI, ¥ 316–¥ 896) and for no adverse reactions in the digestive system is ¥ 396 (95% CI, ¥ 152–¥ 640).

**Table 4:** Patients' trade-off of drug costs.

	WTP^*^^Δ^(95% CI), average ¥ **per month**			
**Attributes**	**Total of patients^a^ (*N* = 151)**	**Monotherapy^b^ (*N* = 77)**	**Two AEDs^b^ (*N* = 58)**	**Three or more AEDs^b^ (*N* = 16)**
**The efficacy of AEDs**				
100% controlled	1,246 (632, 1,861)	2,128 (−213, 4,470)	1,085 (365, 1,752)	346 (52, 639)
75% less	−190 (−354, −26)	−441 (−1,028, 146)	−100 (−287, 86)	4 (−179, 188)
50% less	−1,056 (−1,567, −545)	−1,687 (−3,517, 142)	−957 (−1,572, −343)	−350 (−638, −63)
**Adverse reactions of digestive system**				
0 in 10 people (0%)	396 (152,640)	709 (−152, 1,570)	258 (6, 510)	227 (−21, 476)
1 in 10 people (10%)	211 (54,369)	162 (−164, 488)	340 (72, 608)	39 (−135, 212)
3 in 10 people (30%)	−607 (−929, −286)	−871 (−1,872, 130)	−598 (−1,019, −176)	−266 (−510, −23)
**Duration of side effects in neuropsychic systems**				
None	606 (316,896)	904 (−47, 1,855)	659 (246, 1,073)	65 (−125, 254)
Last for 1 week	−334 (−501, −167)	−487 (−989, 15)	−335 (−554, −116)	−111 (−283, 62)
Last for 4 weeks	−272 (−457, −87)	−417 (−963, 128)	−324 (−600, −48)	46 (−123, 218)
**Effects of AEDs on the fetus**				
3 in 100 people (3%)	1,112 (586, 1,658)	1,837 (−137, 3,812)	911 (338, 1,484)	544 (152, 936)
6 in 100 people (6%)	−125 (−252, 3)	−302 (−699, 95)	−96 (−261, 69)	61 (−119, 241)
10 in 100 people (10%)	−997 (−1,485, −510)	−1,536 (−3,216, 144)	−815 (−1,340, −290)	−605 (−1,046, −164)

* *The calculation of willingness-to-pay is the mean estimate derived from the alternative-specific conditional logit model without interactions; Negative value is the average costs that must be reduced for patients who choose a treatment with the characteristic*.

Δ *$US1 = ¥ 7, £GBP1 = ¥ 9, €EUR1 = ¥ 8, 2020 exchange rate*.

a *Represent the mean WTP of all respondents*.

b *Represent the mean WTP for the current actual AEDs treatment regimens of different patients*.

Then the authors identified the number of AEDs that the respondents were using. As shown in Table 4, the WTP of respondents in various attributes decreases as the number of AED patients increases. For example, patients who are treated with monotherapy are willing to pay ¥ 2,128 to achieve 100% seizure control. However, those who use three or more AEDs are willing to pay ¥ 346 for the same treatment. In general, the result shows that the patients have stronger negative values as the efficacy deteriorates and the risk of side effects increases.

## Discussion

This paper is the first study that uses discrete choice experiments to quantify patients' benefit-risk preferences and their trade-off to treat AEDs in China. It is also the first study to investigate the preference of males with epilepsy for the effect of AEDs on the fetus. The research shows that improving drug efficacy or better controlling seizures and reducing the risk of adverse reactions are the predominant considerations for the patients when choosing their treatments. Among all six attributes observed in the paper, the attribute of AEDs efficacy is the most important one for patients to select the treatment, and that is followed by the effects of AEDs on the fetus. Besides, the digestive system's adverse reaction and the duration of side effects in neuropsychic systems have statistical significance. This result is similar to the earlier literature. Ettinger et al. (16) used DCE in patients and physicians and found that both of them consider seizure control as the most critical factor. Manjunath et al. (15) investigated patient preference for add-on AEDs, and they found that the reduction of seizure frequency was concerned more than that of adverse effects. Unexpectedly, dosing frequency does not significantly influence patients' treatment preferences, which indicates that patients may rely on their current treatment options. Many patients have epilepsy for the average course of more than 10 years, and they need to take AEDs at least twice a day. Therefore, the increase of dosing frequency may have little impact on their quality of life. Besides, the patients' WTP is strongest when it comes to being free from seizure, and the WTP degree is followed when it comes to reducing the risk of fetal abnormality to 3%. This proves the value of improving drug efficacy and reducing the risk of side effects.

Furthermore, the study investigates the impact of personal conditions on treatment preferences. It is found that pregnancy intentions and AEDs treatment regimens significantly affect the patients' preference degree.

Firstly, epilepsy patients preparing for pregnancy have a strong positive preference for reducing the risk of AEDs to the fetus. This finding is consistent with the study of Holmes et al. (17), which investigated the women with productive potential but with generalized or unclassified epilepsy. According to the time of treatment failure in the SNADA study (42), AED is ranked as valproate > topiramate > lamotrigine for generalized or unclassified epilepsy patients. But combined with data and clinical evidence of patient preference, the sequence is reversed, i.e., lamotrigine > topiramate > valproate. Therefore, the treatment preference of patients will vary with individual attributes. For example, in this study, epilepsy patients who have pregnant intentions are more concerned about the effects of drugs on the fetus than about the control of epilepsy.

Secondly, there is no difference between males and females in terms of the preference for AEDs, which means that male patients are also concerned about the effect of drugs on the fetus. As there was no enough evidence to confirm that AEDs taken by male patients who are about to father a baby can cause fetal malformation (35), it's worthwhile to study whether hormonal problems can negatively impact patients' fertility in future work.

Thirdly, patients' WTP decreases significantly as the number of AEDs that they need to take increases. For example, the WTP percentages of patients who use three or more AEDs take in monotherapy patients regarding three factors: acquiring 100% seizure control, no side effects in the digestive system, and no adverse reaction in neuropsychic systems, are 1/6, 1/3, 1/13, respectively.

The first possible reason is that the patients who take three or more drugs are more likely to be drug-resistant. Their relatively large total defined daily dose (DDD) can lead to a heavy economic burden on drugs. Therefore, with the same income, patients taking three or more AEDs have lower WTP on controlling seizures and reducing adverse reactions than those who choose monotherapy. According to the literature reported by Hong et al. (33), the overall average annual spending on epilepsy treatment accounts for more than half of patients' mean yearly income, and the economic burden of patients with drug-resistant epilepsy is approximately eight times that of seizure-free patients. We know the main goal of the AEDs treatment is the total seizure control. However, due to the reduction of WTP in patients with drug-resistant epilepsy, the adverse effects of AEDs may negatively impact the patients' quality of life more than the seizure frequency.

The second possible reason is that monotherapy can help many patients achieve seizure-free after. Some literature show that about 47% of newly diagnosed patients can become seizure-free after the first use of AED, while 13% and 1% of them can be seizure-free after the second and third single drug treatment (43, 44). It can be seen that some patients with monotherapy may have ideal epilepsy control, and they may be willing to spend more money on the following treatments. This result is similar to that of Lloyd et al. (14), which showed that participants who experienced seizures every week have a WTP for £27 per month to achieve 100% seizure control, £38 and £89 for monthly and yearly seizures, respectively. The data means that patients with lower seizure frequency are more likely to pay more to improve seizure control. For drug-resistant epilepsy or refractory epilepsy patient, physicians should pay more attention to their economic burden of drugs in the clinic.

This study shows that DCE is an effective and accurate method for acquiring patient preference for AEDs treatment. It can quantify different treatment attributes and their levels, providing useful information to help researchers understand their importance. Epilepsy is a chronic disease that needs individualized treatment. Understanding the patient treatment preference through DCE and taking it into account when making decisions can facilitate decision sharing between doctors and patients, which in turn can improve HRQOL and therapeutic compliance.

Some limitations should also be highlighted. First, as it was infeasible to include all the treatment attributes in the choice set, this study only selected the six most representative indicators. Therefore, these attributes may not accurately reflect their displayed preferences. For example, major side effect of AEDs such as sedation, cognitive deficits, overweight/obesity, and cardiovascular risks were not listed as a separate attribute. Second, in the choice set of DCEs, patients were required to choose from a pair of hypothetical AEDs, but the choice of real AEDs may reveal different findings. Third, the patients were all recruited from the epilepsy center of tertiary hospitals in China, while those from primary and secondary hospitals are not considered.

## Data Availability Statement

The raw data supporting the conclusions of this article will be made available by the authors, without undue reservation.

## Ethics Statement

The studies involving human participants were reviewed and approved by the Human Research Ethics committee of the First Affiliated Hospital of Wenzhou Medical University. The patients/participants provided their written informed consent to participate in this study.

## Author Contributions

Material preparation, data collection, and analysis were performed by YH, XL, JG, JL, XW, ZZ, and HX. The first draft of the manuscript was written by YH and ZZ. All authors commented on previous versions of the manuscript, contributed to the study conception and design, and read and approved the final manuscript.

## Conflict of Interest

The authors declare that the research was conducted in the absence of any commercial or financial relationships that could be construed as a potential conflict of interest.

## Abbreviations

WTP: willingness-to-pay
AEDs: antiepileptic drugs
DCEs: discrete choice experiments
ADHD: attention-deficit/hyperactivity disorder
LB: low bound
UB: upper bound.
